# Electronic cigarette and smoking paraphernalia point of sale displays: an observational study in England

**DOI:** 10.1136/tobaccocontrol-2020-056314

**Published:** 2021-09-13

**Authors:** Laura A Brocklebank, Anna K M Blackwell, Theresa M Marteau, Katie De-loyde, Richard W Morris, Thomas Burgoine, Alice Hobson, Minna Ventsel, Marcus R Munafo

**Affiliations:** 1 School of Psychological Science, University of Bristol, Bristol, UK; 2 Department of Behavioural Science and Health, University College London, London, UK; 3 Behaviour and Health Research Unit, University of Cambridge, Cambridge, UK; 4 Department of Population Health Sciences, University of Bristol, Bristol, UK; 5 UKCRC Centre for Diet and Activity Research (CEDAR), MRC Epidemiology Unit, University of Cambridge School of Clinical Medicine, Cambridge, UK

**Keywords:** advertising and promotion, electronic nicotine delivery devices, environment, socioeconomic status

## Abstract

**Background:**

Tobacco point of sale (POS) retail displays are banned in many countries, including in England, due in part to evidence linking them to greater susceptibility to smoking in children. There is no equivalent ban on displays of electronic cigarettes (e-cigarettes) or smoking paraphernalia (eg, cigarette lighters) in England, which are often positioned alongside covered tobacco storage units. This observational study describes the visibility and placement of e-cigarette and smoking paraphernalia POS displays in major tobacco retailers in two cities in England to inform future research examining their possible links to susceptibility to tobacco smoking, particularly in children.

**Methods:**

Researchers visited all small- and large-format stores of four supermarket chains and a randomly selected sample of convenience stores, in Bristol and Cambridge. A standardised checklist was used to create a total visibility score for POS displays of (a) e-cigarettes and (b) smoking paraphernalia, plus other measures of visibility and placement. These were described for the total sample and compared between areas of low, medium, and high deprivation using general linear models adjusting for store location and store type.

**Results:**

The visibility checklist was completed in 133 of 166 stores (80% completion rate). Both e-cigarette and smoking paraphernalia POS displays were present in 96% of stores. POS displays were highly visible across all stores: mean (SD) total visibility scores, out of 17, were 14.7 (1.8) for e-cigarettes and 12.7 (1.8) for smoking paraphernalia. There was no clear evidence of differences in visibility by area of deprivation.

**Conclusion:**

E-cigarette and smoking paraphernalia POS displays are near ubiquitous and highly visible in major tobacco retailers in two cities in England. The impact of these displays on tobacco smoking in children and adults is unknown, meriting urgent research to assess their effect on susceptibility to tobacco smoking in children.

## Introduction

Displays of tobacco products at point of sale (POS) in retail stores (eg, cigarettes, loose tobacco, and heated tobacco), which are linked to increased smoking and greater susceptibility to smoking in children,^1 2^ are banned in many countries. Between 2001 and 2016, 20 countries implemented tobacco POS display bans,^3^ including England. Increasingly they are being replaced with tobacco storage units, often positioned alongside tobacco signage and open displays of electronic cigarettes (e-cigarettes) and smoking paraphernalia (eg, cigarette lighters). In 2016, the European Union (EU) Tobacco Products Directive introduced new regulations for e-cigarette retailers,^4^ prohibiting in retail stores: (1) additional imagery that is not of the product; and (2) overly descriptive language that describes products in a way that cannot be objectively substantiated.^5^ However, a ban on e-cigarette POS displays similar to tobacco was not introduced.

A balanced approach to e-cigarette sales is required to promote their use as a smoking cessation aid, while avoiding any adverse impacts on non-smokers of product exposure. As manufacturers adapt to the new EU regulations, it is timely to determine whether the more permissive approach to e-cigarette POS displays could adversely impact youth smoking.^6–9^

E-cigarette POS exposure is associated with vaping,^10–12^ similar to tobacco POS exposure and smoking. Exposure to a combination of tobacco and e-cigarette advertising, including in retail stores, is associated with higher odds of current tobacco use in young people, compared with exposure to tobacco advertising alone.^13^ Examination of potential cross-product associations between e-cigarette exposure and smoking is therefore warranted.

At present, the extent of this potential problem is unknown because the visibility and placement of e-cigarette and smoking paraphernalia POS displays in major tobacco retailers has not been described in detail. The current study addresses this gap to inform future research examining the impact of e-cigarette and smoking paraphernalia POS displays on tobacco smoking, particularly in children. We also examined differences in visibility according to area of deprivation, hypothesising that visibility would be higher in more deprived areas.

## Methods

### Study design

This observational study describes the visibility and placement of e-cigarette and smoking paraphernalia POS displays in major tobacco retailers—small- and large-format supermarkets and convenience stores—in Bristol and Cambridge, England, sampled to include areas varying by deprivation.

### Sample

The sample comprised 166 retail stores within a 10 mile radius of the University of Bristol and the University of Cambridge. This included all small- and large-format stores of four supermarket chains in England (n=88; Bristol 61, Cambridge 27), and a randomly selected sample of convenience stores from the same postcode districts (n=78; Bristol 54, Cambridge 24). Convenience store locations were obtained from 2018 Ordnance Survey Points of Interest data.^14^ Supermarkets and convenience stores were chosen as they account for the majority of the EU tobacco market share (Public Health England, personal communication, November 2019). The initial sample size was based on the DISPLAY study.^15^ Across 96 retail stores, the mean (SD) visibility score (out of 22) was 15.91 (1.82). Accordingly, 131 stores were required to estimate the true mean visibility score to within 2% of its true value, with 95% confidence. Researchers planned to visit 19 additional stores to allow for 14% refusals,^16^ resulting in a planned sample size of 150. The sample was later supplemented with 16 small-format supermarkets, resulting in a final sample of 166.

### Measures

#### Primary outcomes

Visibility of POS displays was assessed using a checklist, adapted from the tobacco visibility tool used in the DISPLAY study,^15^ which provided a total visibility score (range: 0 (low) to 17 (high)) for (a) e-cigarettes and (b) smoking paraphernalia (see online supplemental material).

10.1136/tobaccocontrol-2020-056314.supp1 Supplementary data

#### Secondary outcomes

The checklist also assessed for (a) e-cigarettes and (b) smoking paraphernalia:

1. Number of display units (1/>1)
2. Presence of signage (yes/ no);
3. Presence of visible pricing (yes/no)
4. Size of the display versus tobacco storage unit (smaller (or separate)/same size or larger).

For e-cigarettes only:

1. Presence of promotional material (yes/no).

#### Additional measures

Researchers recorded free-text descriptions for (a) e-cigarettes and (b) smoking paraphernalia:

1. Presence of other types of signage (tobacco, heated tobacco, and/or other)
2. Other products with visible pricing (tobacco, heated tobacco, and/or other)
3. Visibility features (position, size, options, signage, lighting, colours, and/or other)
4. Position of the display versus tobacco storage unit (above, below, adjacent, in front, shared, multiple positions (or separate)).

For e-cigarettes only:

1. Types of promotional material (health, price, flavours, and/or other).

### Procedure

A researcher approached sales assistants or managers for permission to complete the checklist and to photograph the POS display. The photographs were used to resolve any discrepancies between dual ratings, and to confirm visibility scores.

### Data analysis

For each total visibility score, a general linear model was used to assess differences between areas of low, medium, and high deprivation after adjusting for store location (Bristol or Cambridge) and store type (convenience store or supermarket). Data are reported as mean differences (95% CI). As a secondary analysis, an interaction term for deprivation level and store type was added to the model to investigate whether area of deprivation had a different effect in convenience stores than in supermarkets.

For all secondary outcomes, logistic regression was used to assess differences between areas of low, medium, and high deprivation after adjusting for store location and store type. Data are reported as OR (95% CI). Additional measures are presented by deprivation level (online supplemental table S1).

Analyses of both primary outcomes were repeated using a bootstrapping method, using 1000 bootstrap samples, due to deviations from normality in their distributions.^17^ The results were similar (online supplemental table S2).

Further details of the study methods are presented in the pre-registered protocol (osf.io/xks6d).

## Results

### Sample characteristics

The final sample included checklist data for 133 of 166 stores (80%; 11% refused permission, 8% excluded (4% permanently closed, 4% not selling e-cigarettes or smoking paraphernalia)). Included stores had similar characteristics to the total sample (online supplemental table S3). Of the included stores, 71% were in Bristol and 29% were in Cambridge; 43% were convenience stores, 40% were small-format supermarkets, and 17% were large-format supermarkets; 33% were in areas of low deprivation, 48% were in areas of medium deprivation, and 19% were in areas of high deprivation.

### E-cigarette and smoking paraphernalia visibility for all stores

POS displays were highly visible, particularly for e-cigarettes, across all stores. The mean (SD) total visibility score, out of 17, was 14.7 (1.8) for e-cigarettes and 12.7 (1.8) for smoking paraphernalia (table 1). Notable visibility features for both types of POS display were their position, size, and colour.

**Table 1:** Visibility of point-of-sale displays for e-cigarettes and smoking paraphernalia in supermarkets and convenience stores: England, November 2019 to February 2020 (n=133).

	E-cigarettes	Smoking paraphernalia
**Total visibility score (out of 17**) Mean (SD), range	14.7 (1.8), 10–17	12.7 (1.8), 7–17
**Number of display units**		
1 display unit, % (N)	47 (63)	88 (117)
>1 display unit, % (N)	53 (70)	12 (16)
**Presence of signage**		
Present, % (N)	62 (82)	5 (7)
Absent, % (N)	38 (51)	95 (126)
**Presence of visible pricing**		
Present, % (N)	70 (93)	45 (60)
Absent, % (N)	30 (40)	55 (73)
Relative size*		
Larger, % (N)	11 (15)	3 (4)
Same size, % (N)	12 (16)	2 (2)
Smaller, % (N)	74 (99)	93 (123)
Separate, % (N)	2 (3)	3 (4)
**Presence of promotional material**		
Present, % (N)	53 (71)	–
Absent, % (N)	47 (62)	–

Data are reported as mean (SD), range; or percentage (number).

Thirty-one convenience stores from the initial sample were excluded (52% permanently closed; 48% not selling e-cigarettes or smoking paraphernalia). Replacement stores matched by postcode district to the excluded stores were randomly selected from the total list of convenience stores. The visibility checklist was not completed in 15 stores from the initial sample (nine supermarkets and six convenience stores: 40% manager declined; 27% no reason; 13% manager absent; 13% too busy; and 7% manager-perceived unsuitability). A smaller number of supermarkets were located in areas of high deprivation, and refusals resulted in a smaller number of checklists being completed in these supermarkets.

*Compared with the tobacco storage unit.

SD, standard deviation.

The use of multiple display units was more common for e-cigarettes (53%) than for smoking paraphernalia (12%). Signage was present in most stores (62%) for e-cigarettes, but not for smoking paraphernalia (5%). Visible pricing was present in most stores (70%) for e-cigarettes, but in 45% for smoking paraphernalia. In the majority of stores, e-cigarette (74%) and smoking paraphernalia (93%) displays were smaller than the tobacco storage unit, and positioned adjacent to it (49% and 50%, respectively). Finally, 53% of stores had some form of promotional material for e-cigarettes, with the most common types involving price (23%), ease of use (15%), and flavours (14%) (online supplemental table S1).

### Comparing visibility between areas of low, medium, and high deprivation

Contrary to expectation, there was no clear evidence of differences in the visibility of e-cigarette and smoking paraphernalia POS displays between areas of low, medium, and high deprivation after adjusting for store location and store type (table 2). There was also no clear evidence of an interaction between deprivation level and store type (p=0.72 and p=0.82 for the total visibility score for e-cigarettes and smoking paraphernalia, respectively).

**Table 2:** Visibility of point-of-sale displays for e-cigarettes and smoking paraphernalia, by deprivation level: England, November 2019 to February 2020 (N=132*).

	Total visibility score for e-cigarettes			
	Unadjusted mean (SD)		Estimated MD (95% CI)†	P value†
**Deprivation level**				
Low (n=44)	14.84 (1.80)		–	–
Medium (n=63)	14.75 (1.74)		−0.021 (−0.695 to 0.653)	0.951
High (n=25)	14.48 (1.83)		−0.150 (−1.045 to 0.745)	0.741

	Total visibility score for smoking paraphernalia			
	Unadjusted mean (SD)		Estimated MD (95% CI)†	P value†
**Deprivation level**				
Low (n=44)	12.61 (1.88)		–	–
Medium (n=63)	12.54 (1.86)		−0.007 (−0.711 to 0.696)	0.983
High (n=25)	13.08 (1.68)		0.670 (−0.265 to 1.605)	0.159

	Number of display units for e-cigarettes			
	1, % (N)	>1, % (N)	Estimated OR (95% CI)†	P value†
**Deprivation level**				
Low (n=44)	48 (21)	52 (23)	–	–
Medium (n=63)	49 (31)	51 (32)	0.87 (0.39 to 1.93)	0.735
High (n=25)	44 (11)	56 (14)	0.79 (0.27 to 2.27)	0.659

	Number of display units for smoking paraphernalia			
	1, % (N)	>1, % (N)	Estimated OR (95% CI)†	P value†
**Deprivation level**				
Low (n=44)	84 (37)	16 (7)	–	–
Medium (n=63)	89 (56)	11 (7)	0.64 (0.20 to 2.07)	0.643
High (n=25)	92 (23)	8 (2)	0.35 (0.06 to 2.02)	0.352

	Presence of signage for e-cigarettes			
	Absent, % (N)	Present, % (N)	Estimated OR (95% CI)†	P value†
**Deprivation level**				
Low (n=44)	41 (18)	59 (26)	–	–
Medium (n=63)	37 (23)	64 (40)	1.22 (0.55 to 2.71)	0.628
High (n=25)	36 (9)	64 (16)	1.38 (0.47 to 4.02)	0.555

	Presence of signage for smoking paraphernalia			
	Absent, % (N)	Present, % (N)	Estimated OR (95% CI)†	P value†
**Deprivation level**				
Low (n=44)	91 (40)	9 (4)	–	–
Medium (n=63)	98 (62)	2 (1)	0.15 (0.02 to 1.43)	0.100
High (n=25)	92 (23)	8 (2)	0.83 (0.13 to 5.46)	0.846

	Presence of visible pricing for e-cigarettes			
	Absent, % (N)	Present, % (N)	Estimated OR (95% CI)†	P value†
**Deprivation level**				
Low (n=44)	36 (16)	64 (28)	–	–
Medium (n=63)	32 (20)	69 (43)	0.68 (0.15 to 3.16)	0.622
High (n=25)	16 (4)	84 (21)	0.42 (0.06 to 3.07)	0.392

	Presence of visible pricing for smoking paraphernalia			
	Absent, % (N)	Present, % (N)	Estimated OR (95% CI)†	P value†
**Deprivation level**				
Low (n=44)	59 (26)	41 (18)	–	–
Medium (n=63)	56 (35)	44 (28)	1.07 (0.32 to 3.58)	0.909
High (n=25)	48 (12)	52 (13)	1.42 (0.34 to 5.86)	0.632

	Relative size of the display unit for e-cigarettes‡			
	<Tobacco, % (N)	≥Tobacco, % (N)	Estimated OR (95% CI)†	P value†
**Deprivation level**				
Low (n=44)	71 (31)	30 (13)	–	–
Medium (n=63)	84 (53)	16 (10)	0.33 (0.12 to 0.92)	0.034
High (n=25)	68 (17)	32 (8)	0.53 (0.16 to 1.74)	0.298

	Relative size of the display unit for smoking paraphernalia‡			
	<Tobacco, % (N)	≥Tobacco, % (N)	Estimated OR (95% CI)†	P value†
**Deprivation level**				
Low (n=44)	98 (43)	2 (1)	–	–
Medium (n=63)	97 (61)	3 (2)	1.12 (0.90 to 13.44)	0.927
High (n=25)	88 (22)	12 (3)	2.84 (0.26 to 31.00)	0.391

	Presence of promotional material for e-cigarettes			
	Absent, % (N)	Present, % (N)	Estimated OR (95% CI)†	P value†
**Deprivation level**				
Low (n=44)	59 (26)	41 (18)	–	–
Medium (n=63)	49 (31)	51 (32)	1.39 (0.52 to 3.67)	0.512
High (n=25)	20 (5)	80 (20)	2.71 (0.71 to 10.29)	0.143

Deprivation level of lower super output area (LSOA) was from the 2019 Index of Multiple Deprivation (IMD) deciles^19^: (1) low (8–10); (2) medium (4–7); and (3) high (1–3). It was assumed that the variance between deprivation levels would be 25% of the residual variance, equivalent to assuming a minimum effect size of d=0.5. It was estimated that at least nine stores would be required in each group to have 90% power to detect an effect size of this magnitude or greater when comparing the visibility of POS displays between deprivation levels after adjusting for store type.

*132 rather than 133 because one small-format supermarket in Bristol had missing IMD data.

†Models were adjusted for store location (Bristol or Cambridge) and store type (convenience store or supermarket).

‡Compared with the tobacco storage unit: <, smaller than the tobacco storage unit (or separate); or ≥, the same size or larger than the tobacco storage unit.

95% CI, 95% confidence interval; IMD, Index of Multiple Deprivation; MD, mean difference; OR, odds ratio; SD, standard deviation.

## Discussion

E-cigarette and smoking paraphernalia POS displays were ubiquitous and highly visible across all supermarkets and convenience stores in two cities in England, with no clear evidence of differences by area of deprivation.

The current study builds on previous work on the prevalence of e-cigarette POS displays in Scotland^18^ to examine the visibility of e-cigarette and smoking paraphernalia POS displays in England. Before implementation of the tobacco POS display ban, tobacco products and tobacco storage units were highly visible across all tobacco retailers in Scotland.^15^ Product visibility did not differ by area of deprivation, but storage unit visibility was higher in areas of high deprivation than in areas of low deprivation. Implementation of the tobacco POS display ban drastically reduced product visibility, and to a lesser extent storage unit visibility. It also eliminated the socioeconomic difference in storage unit visibility.

Exposure to e-cigarette POS displays is associated with increased vaping,^10–12^ but any impact of e-cigarette displays on smoking is unknown. Further research should examine whether the visibility of e-cigarette and smoking paraphernalia POS displays has an impact on smoking, thereby undermining the effectiveness of the tobacco POS display ban. In addition, if exposure to e-cigarette or smoking paraphernalia displays is associated with susceptibility to tobacco smoking in children, policymakers may want to expand the current ban.

The size and position of e-cigarette POS displays were the two most common features of product visibility recorded by researchers in the majority of both convenience stores (77% and 97%, respectively) and large-format supermarkets (87% and 96%, respectively). In convenience stores, multiple displays were generally found on the counter at the POS, whereas in large-format supermarkets, large displays were generally found spanning the full length behind separate tobacco kiosks. The availability of many colourful product options were also key features of e-cigarette visibility in approximately three-quarters of both convenience stores and large-format supermarkets. In contrast, the use of e-cigarette-specific signage and display lighting were more common features found in large-format supermarkets (74% and 52%, respectively) compared with convenience stores (56% and 18%, respectively). We did not plan to directly compare visibility features by store type, and these exploratory findings should be interpreted with caution. However, these differences are potentially important—they may appeal to, or deliberately target, different audiences, including young non-smokers and adult smokers. Future studies should consider the impact of different display features on different groups.

### Strengths and limitations

To our knowledge, this is the first study to describe the visibility and placement of e-cigarette and smoking paraphernalia POS displays in major tobacco retailers. The use of a standardised measure of visibility—adapted from a comprehensive tobacco visibility tool^15^—is also a strength. Finally, the use of overt measurement meant that observer recall was supplemented with photographs in the majority (77%) of stores, increasing accuracy.

Including stores from only two cities may limit generalisability of the findings, particularly for convenience stores, given that supermarket chains tend to have standardised layouts. Furthermore, store type may be a mediator, rather than a confounder, of the association between area deprivation and visibility of e-cigarette and smoking paraphernalia POS displays due to store types varying by deprivation level. However, minimally adjusted analyses, which included only deprivation level and store location (and not store type), gave very similar results.

## Conclusion

E-cigarette and smoking paraphernalia POS displays are near ubiquitous and highly visible in supermarkets and convenience stores in two cities in England. The impact of these displays on tobacco smoking in children and adults is unknown, meriting urgent research to assess their effect on susceptibility to tobacco smoking in children.

**What this paper adds**

- Tobacco point of sale (POS) retail displays are banned in many countries, including in England, due in part to evidence linking them to increased smoking and greater susceptibility to smoking in children.
- There is no equivalent ban on displays of electronic cigarettes (e-cigarettes) or smoking paraphernalia (eg, cigarette lighters) in England, often positioned alongside covered tobacco storage units.
- This observational study is the first, to our knowledge, to describe the visibility and placement of e-cigarette and smoking paraphernalia POS displays in major tobacco retailers.
- Our results show that e-cigarette and smoking paraphernalia POS displays are near ubiquitous and highly visible in supermarkets and convenience stores in two cities in England.
- The high frequency and visibility of these displays could be undermining the effectiveness of the tobacco POS display ban. Their impact on smoking in children merits urgent attention.

## Data Availability

The datasets generated and analysed during the current study are available at the University of Bristol data repository, data.bris, at https://doi.org/10.5523/bris.2u6wn87v1hc5a2oltkhmdlfnr3.
