# Supplementary material for: Electronic cigarette and smoking paraphernalia point of sale displays: an observational study in England
Source: Tob Control. 2021 Sep 13;31(e2):e201–6. doi: 10.1136/tobaccocontrol-2020-056314 (PMC9726958; doi:10.1136/tobaccocontrol-2020-056314)
Supplement: Supplementary data [file tobaccocontrol-2020-056314supp001.pdf]

Electronic cigarette and smoking paraphernalia point of sale displays

SUPPLEMENTARY MATERIAL

Supplementary Material. Visibility checklist**.**

1. Date Click or tap here to enter text.
2. Researcher initials Click or tap here to enter text.
3. Store ID Click or tap here to enter text.
4. Type of store
    a. Large supermarket – Tesco ☐
    b. Large supermarket – Sainsbury's ☐
    c. Large supermarket – Asda ☐
    d. Small supermarket – Tesco ☐
    e. Small supermarket – Sainsbury's ☐
    f. Small supermarket – Co-op ☐
    g. Convenience store ☐
5. Permission to complete checklist *(only ask for the manager if met with uncertainty or reluctance)*
    a. Yes ☐
    b. No ☐ - please give reason Click or tap here to enter text.
6. Permission to take photographs *(only ask for the manager if met with uncertainty or reluctance)*
    a. Yes ☐
    b. No ☐ - please give reason Click or tap here to enter text.
7. Which of the following products are available? *(ask about missing products as they may be hidden)*
    a. Cigarettes ☐
    b. Loose tobacco ☐
    c. Heated tobacco ☐
    d. Lighters ☐
    e. Matches ☐
    f. Papers ☐
    g. Filters ☐
    h. E-cigarettes ☐
    i. E-liquids ☐
    j. Other ☐ - please describe Click or tap here to enter text.

Brocklebank LA, *et al. Tob Control* 2022; 31:e201–e206. doi: 10.1136/tobaccocontrol-2020-056314

Electronic cigarette and smoking paraphernalia point of sale displays

E-cigarettes

Total visibility score (out of 17)

1. How visible are the e-cigarettes?
   a. Full visibility: on permanent display *(2 points)* ☐
   b. Some visibility: concealed within a public facing storage unit *(1 point)* ☐
   c. Zero visibility: stored out of sight *(0 points)* ☐
2. Is the display unit positioned on or behind the counter? *(i.e., out of customer reach)*
   a. Yes *(1 point)* ☐
   b. No *(0 points)* ☐ - please give details Click or tap here to enter text.
   c. Out of sight *(0 points)* ☐
3. Is the display unit positioned at customer eye level? *(i.e., all or part of the unit is above the counter)*
   a. Yes *(1 point)* ☐
   b. No *(0 points)* ☐ - please give details Click or tap here to enter text.
   c. Out of sight *(0 points)* ☐
4. Is the display unit within arms-reach of the shop assistant serving the customer? *(i.e., no more than 2 steps distance from the nearest till-point)*
   a. Yes *(1 point)* ☐
   b. No *(0 points)* ☐ - please give details Click or tap here to enter text.
   c. Out of sight *(0 points)* ☐
5. From what sales counter are e-cigarettes purchased from?
   a. The main sales counter where most other goods are purchased *(5 points)* ☐
   b. A separate tobacco counter or kiosk ☐
   c. Both *(5 points)* ☐
6. If customers purchase e-cigarettes from a separate counter, where is it positioned?
   a. Customers pass in front of the counter when entering and leaving the store *(4 points)* ☐
   b. Customers pass in front of the counter either when entering or leaving the store *(3 points)* ☐
   c. The counter is in direct line of sight of half or more of the store's service tills *(2 points)* ☐
   d. The counter is in direct line of sight of less than half of the store's service tills *(1 point)* ☐
   e. N/A ☐
7. How conspicuous is the display unit?
   a. 0 (not visible) *(0 points)* ☐
   b. 1 (low visibility) *(1 point)* ☐
   c. 2 *(2 points)* ☐
   d. 3 *(3 points)* ☐
   e. 4 *(4 points)* ☐
   f. 5 (high visibility) *(5 points)* ☐

Brocklebank LA, *et al. Tob Control* 2022; 31:e201–e206. doi: 10.1136/tobaccocontrol-2020-056314

Electronic cigarette and smoking paraphernalia point of sale displays

8. What about the display unit makes it more or less conspicuous?
    a. Position ☐ Click or tap here to enter text.
    b. Size ☐ Click or tap here to enter text.
    c. Options ☐ Click or tap here to enter text.
    d. Signage ☐ Click or tap here to enter text.
    e. Lighting ☐ Click or tap here to enter text.
    f. Colours ☐ Click or tap here to enter text.
    g. Other ☐ Click or tap here to enter text.
9. Is the display unit visible from outside the store *or is there any external signage? (i.e., is the display unit visible if you stand still outside the store and look in all possible directions?)*
    a. Visible from outside the store *(1 point)* ☐
    b. External signage (1 point) ☐
    c. Visible from outside the store and external signage (1 point) ☐
    d. No *(0 points)* ☐
10. Is the display unit visible from the store's main entrance? *(i.e., is the display unit visible if you stand still at the store's main entrance and look in all possible directions?)*
    a. Yes (1 point) ☐
    b. No *(0 points)* ☐

Electronic cigarette and smoking paraphernalia point of sale displays

Additional measures

1. Number of display units Click or tap here to enter text.
2. Is there any signage on the display unit designed to indicate that any of the following products are on sale?
    a. Tobacco ☐
    b. Heated tobacco ☐
    c. E-cigarettes ☐
    d. Smoking paraphernalia ☐
    e. Other *(e.g., lottery)* ☐ - please describe Click or tap here to enter text.
    f. No signage ☐
3. Is there a price list / tag for any of the following products?
    a. Tobacco ☐
    b. Heated tobacco ☐
    c. E-cigarettes ☐
    d. Smoking paraphernalia ☐
    e. Other ☐ - please describe
    f. No price lists / tags ☐
4. Are there any promotional messages for e-cigarettes on the display unit?
    a. Health / smoking cessation ☐
    b. Price ☐
    c. Flavours ☐
    d. Other ☐ - please describe Click or tap here to enter text.
    e. No promotional messages ☐
5. Is the display unit for e-cigarettes near the storage unit for tobacco products?
    a. Yes ☐
    b. No ☐ - please describe Click or tap here to enter text.
6. If near, where is the display unit for e-cigarettes in relation to the storage unit for tobacco products?
    a. Above ☐
    b. Below ☐
    c. Adjacent ☐
    d. In front ☐
    e. Together / shared unit *(this includes a cut-out)* ☐
    f. N/A ☐
7. If near, how does the size of the display unit for e-cigarettes compare to the size of the storage unit for tobacco products?
    a. Smaller ☐
    b. Larger ☐
    c. Same size ☐
    d. N/A ☐
8. Any other comments Click or tap here to enter text.

Brocklebank LA, *et al. Tob Control* 2022; 31:e201–e206. doi: 10.1136/tobaccocontrol-2020-056314

Electronic cigarette and smoking paraphernalia point of sale displays

Smoking paraphernalia

Total visibility score (out of 17)

1. How visible are the smoking paraphernalia?
    a. Full visibility: on permanent display *(2 points)* ☐
    b. Some visibility: concealed within a public facing storage unit *(1 point)* ☐
    c. Zero visibility: stored out of sight *(0 points)* ☐
2. Is the display unit positioned on or behind the counter? *(i.e., out of customer reach)*
    a. Yes *(1 point)* ☐
    b. No *(0 points)* ☐ - please give details Click or tap here to enter text.
    c. Out of sight *(0 points)* ☐
3. Is the display unit positioned at customer eye level? *(i.e., all or part of the unit is above the counter)*
    a. Yes *(1 point)* ☐
    b. No *(0 points)* ☐ - please give details Click or tap here to enter text.
    c. Out of sight *(0 points)* ☐
4. Is the display unit within arms-reach of the shop assistant serving the customer? *(i.e., no more than 2 steps distance from the nearest till-point)*
    a. Yes *(1 point)* ☐
    b. No *(0 points)* ☐ - please give details Click or tap here to enter text.
    c. Out of sight *(0 points)* ☐
5. From what sales counter are smoking paraphernalia purchased from?
    a. The main sales counter where most other goods are purchased *(5 points)* ☐
    b. A separate tobacco counter or kiosk ☐
    c. Both *(5 points)* ☐
6. If customers purchase smoking paraphernalia from a separate counter, where is it positioned?
    a. Customers pass in front of the counter when entering and leaving the store *(4 points)* ☐
    b. Customers pass in front of the counter either when entering or leaving the store *(3 points)* ☐
    c. The counter is in direct line of sight of half or more of the store's service tills *(2 points)* ☐
    d. The counter is in direct line of sight of less than half of the store's service tills *(1 point)* ☐
    e. N/A ☐
7. How conspicuous is the display unit?
    a. 0 (not visible) *(0 points)* ☐
    b. 1 (low visibility) *(1 point)* ☐
    c. 2 *(2 points)* ☐
    d. 3 *(3 points)* ☐
    e. 4 *(4 points)* ☐
    f. 5 (high visibility) *(5 points)* ☐

Electronic cigarette and smoking paraphernalia point of sale displays

8. What about the display unit makes it more or less conspicuous?
    a. Position ☐ Click or tap here to enter text.
    b. Size ☐ Click or tap here to enter text.
    c. Options ☐ Click or tap here to enter text.
    d. Signage ☐ Click or tap here to enter text.
    e. Lighting ☐ Click or tap here to enter text.
    f. Colours ☐ Click or tap here to enter text.
    g. Other ☐ Click or tap here to enter text.
9. Is the display unit visible from outside the store *or is there any external signage? (i.e., is the display unit visible if you stand still outside the store and look in all possible directions?)*
    a. Visible from outside the store *(1 point)* ☐
    b. External signage (1 point) ☐
    c. Visible from outside the store and external signage (1 point) ☐
    d. No *(0 points)* ☐
10. Is the display unit visible from the store's main entrance? *(i.e., is the display unit visible if you stand still at the store's main entrance and look in all possible directions?)*
    a. Yes *(1 point)* ☐
    b. No *(0 points)* ☐

Brocklebank LA, *et al. Tob Control* 2022; 31:e201–e206. doi: 10.1136/tobaccocontrol-2020-056314

Electronic cigarette and smoking paraphernalia point of sale displays

Additional measures

1. Number of display units Click or tap here to enter text.
2. Is the display unit for smoking paraphernalia near the storage unit for tobacco products?
    a. Yes ☐
    b. No ☐ - please describe Click or tap here to enter text.
3. If near, where is the display unit for smoking paraphernalia in relation to the storage unit for tobacco products?
    a. Above ☐
    b. Below ☐
    c. Adjacent ☐
    d. In front ☐
    e. Together / shared unit *(this includes a cut-out)* ☐
    f. N/A ☐
4. If near, how does the size of the display unit for smoking paraphernalia compare to the size of the storage unit for tobacco products?
    a. Smaller ☐
    b. Larger ☐
    c. Same size ☐
    d. N/A ☐

Any other comments Click or tap here to enter text.

Brocklebank LA, *et al. Tob Control* 2022; 31:e201–e206. doi: 10.1136/tobaccocontrol-2020-056314

Electronic cigarette and smoking paraphernalia point of sale displays

Adaptations from the tobacco visibility tool used in the DISPLAY study (1)

Total visibility score

Question 1 (product visibility): Taken from the DISPLAY study but simplified from five to three options. The middle three options were combined into one option for "some visibility".

Questions 2-4 (proximity indicators): Three (of five possible) proximity indicators were taken directly from the DISPLAY study. The fourth indicator – "parallel to the counter" – was excluded as it was deemed to have no added value. The fifth indicator – "generic signage" – was excluded from the total visibility score but captured under additional measures.

Questions 5-6 (proximity of the sales counter to customer traffic): Taken directly from the DISPLAY study.

Questions 7-8 (conspicuousness score): Question 7 (conspicuousness score) was taken directly from the DISPLAY study. Question 8 was added by the researchers to give context to the subjective conspicuousness score, but was not included in the total visibility score

Questions 9-10 (external visibility): Taken directly from the DISPLAY study.

Size: Due to multiple display units, particularly for e-cigarettes, size was assessed by counting the number of separate display units rather than by counting the number of rows and columns (as in the DISPLAY study). Size was also assessed through comparison with the tobacco storage unit. These measures of size were not included in the total visibility score.

Additional measures

All additional measures were added by the researchers based on potential defining characteristics of the e-cigarette and smoking paraphernalia POS displays.

Assessing inter-rater reliability

The first 16 stores (Bristol – 11, Cambridge – 5) were rated independently by two researchers to assess inter-rater reliability. The mean (standard deviation [SD]) of the differences for total visibility score (which were positive when Observer 1's score was higher, and negative when Observer 2's score was higher) were calculated for both e-cigarettes (Bristol – 0.09 [1.04], Cambridge – 0.20 [0.45]) and smoking paraphernalia (Bristol – 0.45 [1.44], Cambridge – 0.60 [1.52])[2]. The remaining stores were rated by one researcher only.

Electronic cigarette and smoking paraphernalia point of sale displays

Supplementary Table S1. Describing the visibility of point of sale (POS) displays of (a) e-cigarettes, (b) smoking paraphernalia, and (c) tobacco products (for comparison) for all stores and by area of deprivation: additional measures (N = 132[a]).

		Total (N = 132)	Deprivation level		
			Low (N = 44)	Medium (N = 63)	High (N = 25)
**Presence of signage**	Tobacco, % (N)	91 (120)	91 (40)	92 (58)	88 (22)
	Heated tobacco, % (N)	17 (23)	14 (6)	18 (11)	24 (6)
	Other – related[b], % (N)	18 (24)	18 (8)	16 (10)	24 (6)
	Other – unrelated[c], % (N)	55 (72)	64 (28)	54 (34)	40 (10)
**Presence of visible pricing**	Tobacco, % (N)	21 (27)	21 (9)	22 (14)	16 (4)
	Heated tobacco, % (N)	1 (1)	2 (1)	0 (0)	0 (0)
**Presence of promotional material for e-cigarettes**	Health, % (N)	5 (6)	7 (3)	2 (1)	8 (2)
	Price, % (N)	23 (30)	16 (7)	21 (13)	40 (10)
	Flavours, % (N)	14 (19)	11 (5)	13 (8)	24 (6)
	Ease of use, % (N)	15 (20)	5 (2)	18 (11)	28 (7)
	Social acceptability, % (N)	2 (3)	0 (0)	3 (2)	4 (1)
	Novelty, % (N)	4 (5)	2 (1)	3 (2)	8 (2)
	Informative, % (N)	3 (4)	7 (3)	2 (1)	0 (0)
	Image, % (N)	5 (6)	9 (4)	2 (1)	4 (1)
	Generic, % (N)	12 (16)	11 (5)	11 (7)	16 (4)
**Presence of visibility features for e-cigarettes**	Position, % (N)	98 (129)	100 (44)	97 (61)	96 (24)
	Size, % (N)	83 (110)	84 (37)	83 (52)	84 (21)
	Options, % (N)	66 (87)	71 (31)	59 (37)	76 (19)
	Signage, % (N)	57 (75)	57 (25)	54 (34)	64 (16)
	Lighting, % (N)	22 (29)	16 (7)	24 (15)	28 (7)
	Colours, % (N)	77 (102)	77 (34)	79 (50)	72 (18)
**Presence of visibility features for smoking paraphernalia**	Position, % (N)	96 (127)	96 (42)	95 (60)	100 (25)
	Size, % (N)	77 (102)	75 (33)	79 (50)	76 (19)
	Options, % (N)	9 (12)	11 (5)	8 (5)	8 (2)
	Signage, % (N)	6 (8)	11 (5)	0 (0)	12 (3)
	Lighting, % (N)	10 (13)	11 (5)	10 (6)	8 (2)
	Colours, % (N)	77 (102)	77 (34)	79 (50)	72 (18)

Brocklebank LA, *et al. Tob Control* 2022; 31:e201–e206. doi: 10.1136/tobaccocontrol-2020-056314

Electronic cigarette and smoking paraphernalia point of sale displays

**Relative position[d] for e-cigarettes**	Above, % (N)	13 (17)	14 (6)	11 (7)	16 (4)
	Below, % (N)	2 (2)	0 (0)	2 (1)	4 (1)
	Adjacent, % (N)	49 (64)	48 (21)	54 (34)	36 (9)
	In front, % (N)	17 (23)	11 (5)	18 (11)	28 (7)
	Shared, % (N)	1 (1)	0 (0)	2 (1)	0 (0)
	Multiple positions, % (N)	17 (22)	23 (10)	13 (8)	16 (4)
	Separate, % (N)	2 (3)	5 (2)	2 (1)	0 (0)
**Relative position[d] for smoking paraphernalia**	Above, % (N)	11 (15)	11 (5)	8 (5)	20 (5)
	Below, % (N)	12 (16)	7 (3)	14 (9)	16 (4)
	Adjacent, % (N)	50 (66)	50 (22)	54 (34)	40 (10)
	In front, % (N)	3 (4)	7 (3)	2 (1)	0 (0)
	Shared, % (N)	14 (19)	18 (8)	13 (8)	12 (3)
	Multiple positions, % (N)	6 (8)	2 (1)	8 (5)	8 (2)
	Separate, % (N)	3 (4)	5 (2)	2 (1)	4 (1)

Deprivation level of lower super output area (LSOA) of store location, using 2019 Index of Multiple Deprivation (IMD) data: low (IMD deciles 8-10), medium (IMD deciles 4-7) or high (IMD deciles 1-3) (3).

[a] 132 rather than 133 because one small-format supermarket in Bristol had missing IMD data

[b] Other types of signage related to tobacco. For example, cannabidiol (CBD), cigars and Nordic Spirit (i.e., nicotine pouches).

[c] Other types of signage unrelated to tobacco. For example, lottery, stamps and mobile top-up.

[d] Compared to the tobacco storage unit.

Brocklebank LA, *et al. Tob Control* 2022; 31:e201–e206. doi: 10.1136/tobaccocontrol-2020-056314

Electronic cigarette and smoking paraphernalia point of sale displays

Supplementary Table S2. Comparing the visibility of point of sale (POS) displays of (a) e-cigarettes and (b) smoking paraphernalia between areas of low, medium and high deprivation (N = 132[a]): bootstrap *p* values and 95% confidence intervals of the mean differences, from 1000 bootstrap samples.

	**Total visibility score for e-cigarettes**			
	**Primary analysis**		**Bootstrap values**	
	Estimated MD (95% CI)*	P value*	95% CI of the MD*	P value*
**Deprivation level**				
Low (N = 44)	-	-	-	-
Medium (N = 63)	-0.021 (-0.695, 0.653)	0.951	-0.675, 0.667	0.966
High (N = 25)	-0.150 (-1.045, 0.745)	0.741	-1.055, 0.759	0.756
	**Total visibility score for smoking paraphernalia**			
	**Primary analysis**		**Bootstrap values**	
	Estimated MD (95% CI)*	P value*	95% CI of the MD*	P value*
**Deprivation level**				
Low (N = 44)	-	-	-	-
Medium (N = 63)	-0.007 (-0.711, 0.696)	0.983	-0.728, 0.653	0.978
High (N = 25)	0.670 (-0.265, 1.605)	0.159	-0.206, 1.546	0.137

Deprivation level of lower super output area (LSOA) of store location, using 2019 Index of Multiple Deprivation (IMD) data: low (IMD deciles 8-10), medium (IMD deciles 4-7) or high (IMD deciles 1-3) (3).

* All models were adjusted for store location (Bristol or Cambridge) and store type (convenience store or supermarket).

[a] 132 rather than 133 because one small-format supermarket in Bristol had missing IMD data.

SD, standard deviation; MD, mean difference; 95% CI, 95% confidence interval

Brocklebank LA, *et al. Tob Control* 2022; 31:e201–e206. doi: 10.1136/tobaccocontrol-2020-056314

Electronic cigarette and smoking paraphernalia point of sale displays

Supplementary Table S3. Sample characteristics (N = 166).

		**Included (N = 133)**		**Refused (N = 19)**		**Excluded (N = 14)**		**Total (N = 166)**	
		N	%	N	%	%	%	N	%
**Store location**	Bristol	95	71	11	58	9	64	115	69
	Cambridge	38	29	8	42	5	36	51	31
	**Total**	**133**	**100**	**19**	**100**	**14**	**100**	**166**	**100**
**Store type**	Convenience store	57	43	8	42	13	93	78	47
	Small-format supermarket	53	40	9	47	1	7	63	38
	Large-format supermarket	23	17	2	11	0	0	25	15
	**Total**	**133**	**100**	**19**	**100**	**14**	**100**	**166**	**100**
**Deprivation level**	Low	44	33	7	37	6	43	57	35
	Medium	63	48	9	47	4	29	76	46
	High	25	19	3	16	4	29	32	19
	**Total**	**132[a]**	**100**	**19**	**100**	**14**	**100**	**165[a]**	**100**
**Deprivation level x Store type**	Low convenience store	16	12	3	16	5	36	24	15
	Medium convenience store	25	19	4	21	4	29	33	20
	High convenience store	16	12	1	5	4	29	21	13
	Low supermarket	28	21	4	21	1	7	33	20
	Medium supermarket	38	29	5	26	0	0	43	26
	High supermarket	9	7	2	11	0	0	11	7
	**Total**	**132[a]**	**100**	**19**	**100**	**14**	**100**	**165[a]**	**100**

Deprivation level of lower super output area (LSOA) of store location, using 2019 Index of Multiple Deprivation (IMD) data: low (IMD deciles 8-10), medium (IMD deciles 4-7) or high (IMD deciles 1-3) (3).

[a] 132 rather than 133 (or 165 rather than 166) because one small-format supermarket in Bristol had missing IMD data.

Electronic cigarette and smoking paraphernalia point of sale displays

References

1. Eadie D, Best CS, Stead M, et al. Assessing change in tobacco visibility at point-of-sale following a display ban. *Tobacco Regulatory Science* 2018; **4**(3): 10-28
2. Bland JM, Altman DG. Statistical methods for assessing agreement between two methods of clinical measurement. *Lancet* 1986; **1**(8476): 307-10
3. Ministry of Housing, Communities & Local Government. English indices of deprivation 2019 [Available from: http://imd-by-postcode.opendatacommunities.org/imd/2019]

Brocklebank LA, *et al. Tob Control* 2022; 31:e201–e206. doi: 10.1136/tobaccocontrol-2020-056314
